# Concomitant Pulmonary and Tonsillar Tuberculosis

**DOI:** 10.7759/cureus.114987

**Published:** 2026-08-22

**Authors:** Mouad Al Moudni, Rajae Azzeddine, Asmaa Jniene, Mustapha Elftouh, Leila Achachi

**Affiliations:** 1 Department of Pulmonology, Ibn Sina University Hospital Center, Rabat, MAR; 2 Department of Physiology, Faculty of Medicine and Pharmacy, Mohammed V University, Rabat, MAR

**Keywords:** case report, extrapulmonary tuberculosis, genexpert mtb/rif, pulmonary tuberculosis, tonsillar tuberculosis

## Abstract

We report the case of a 54-year-old man presenting with concomitant pulmonary and tonsillar tuberculosis. The coexistence of these two localizations poses a diagnostic challenge because of the nonspecific clinical manifestations and the potential confusion with malignant diseases.

The diagnosis of pulmonary tuberculosis was confirmed by positive sputum GeneXpert MTB/RIF, while histopathological examination of the tonsillar lesion revealed non-caseating granulomatous inflammation, with no evidence of malignancy. The patient received the standard anti-tuberculosis therapy for six months, with complete clinical improvement and resolution of the pulmonary lesions at follow-up.

This case highlights the importance of considering tuberculosis in patients presenting with concurrent pulmonary and tonsillar lesions while maintaining suspicion for malignancy because of the overlapping clinical presentation. Early recognition is essential to ensure prompt diagnosis and appropriate management.

## Introduction

Tuberculosis remains a major public health concern worldwide and continues to represent a significant burden in endemic countries such as Morocco, where the World Health Organization estimates an incidence of approximately 97 cases per 100,000 population [1]. Although pulmonary tuberculosis is the most common presentation, extrapulmonary tuberculosis accounts for approximately 15-20% of all cases and may involve virtually any organ [1,2].

Tuberculosis of the upper aerodigestive tract is uncommon, and tonsillar involvement represents one of its rarest manifestations. Tonsillar tuberculosis is often associated with pulmonary disease and may result from the direct inoculation of the oropharyngeal mucosa by infected respiratory secretions. However, hematogenous or lymphatic dissemination may also contribute to tonsillar involvement. The rarity of this condition has been attributed to the protective effects of saliva, the resistance of the stratified squamous epithelium, and the normal oral microbial flora [3,4].

The clinical presentation is often nonspecific, with symptoms such as odynophagia, dysphagia, or unilateral tonsillar enlargement frequently mimicking oropharyngeal malignancy. Consequently, diagnosis may be delayed, and histopathological examination is often required to establish the diagnosis [4].

The coexistence of pulmonary and tonsillar tuberculosis has rarely been reported in the literature. We present this case to emphasize the diagnostic challenges, discuss the differential diagnosis, and highlight the importance of early recognition and appropriate therapeutic management.

## Case presentation

A 54-year-old man, with no previous history of tuberculosis or recent contact with a patient diagnosed with tuberculosis, was referred to our department for a three-month history of productive cough with mucoid sputum. He was an active smoker with a cumulative tobacco exposure of 30 pack-years. His symptoms had progressively worsened and were associated with constitutional manifestations, including an 8-kg weight loss over two months and odynophagia.

On admission, the patient was hemodynamically and respiratorily stable, with an Eastern Cooperative Oncology Group (ECOG) performance status of 0. Physical examination revealed bilateral rhonchi on pulmonary auscultation, whereas the remainder of the clinical examination was unremarkable.

**Figure 1:**
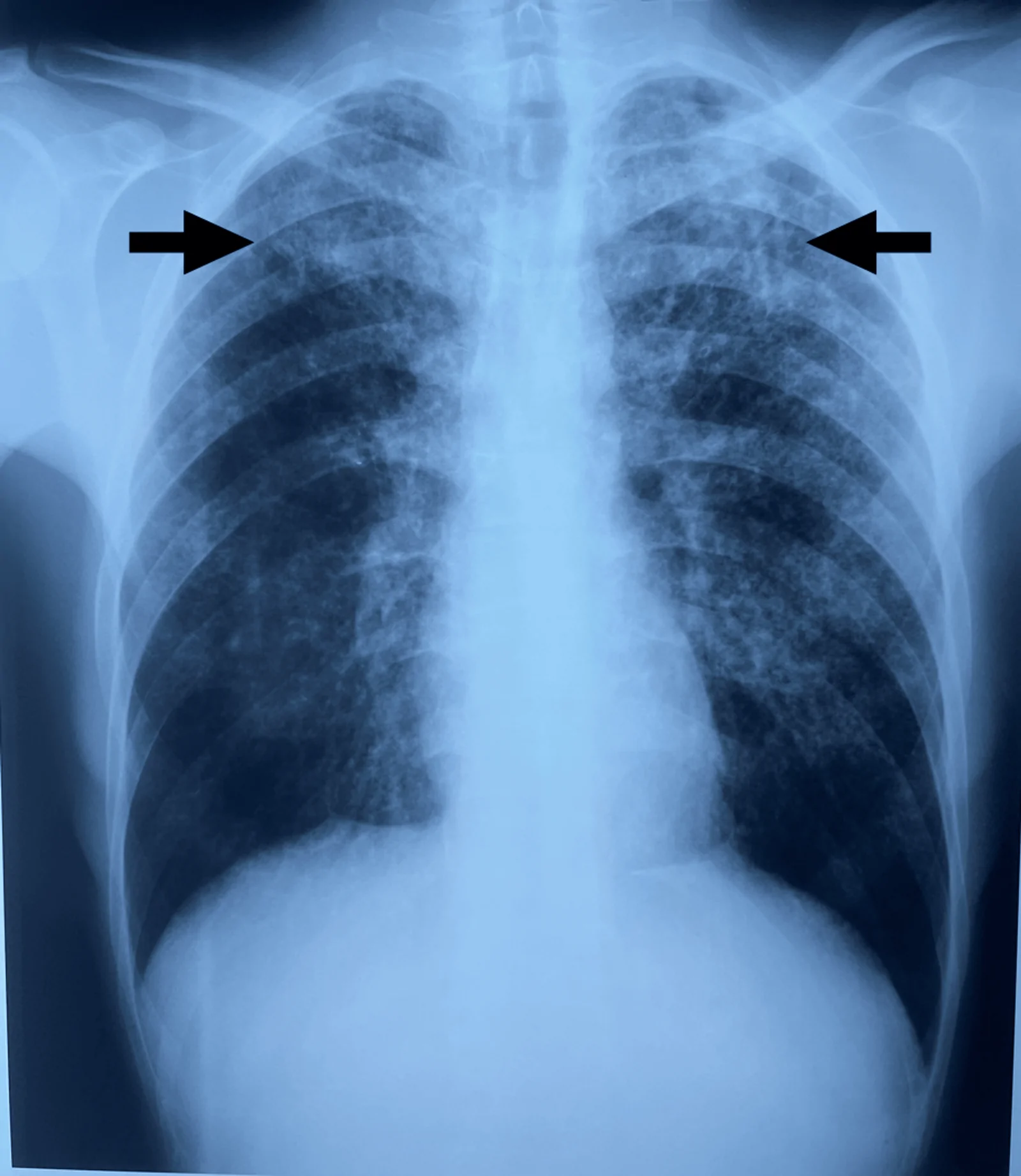
Chest radiograph showing bilateral upper-lobe-predominant infiltrative opacities with suspected cavitary changes

A chest radiograph demonstrated bilateral upper-lobe infiltrative lesions associated with cavitary opacities (Figure 1). Based on these radiological findings and the clinical presentation, pulmonary tuberculosis was considered the leading diagnosis; however, an underlying pulmonary malignancy could not initially be excluded.

**Figure 2:**
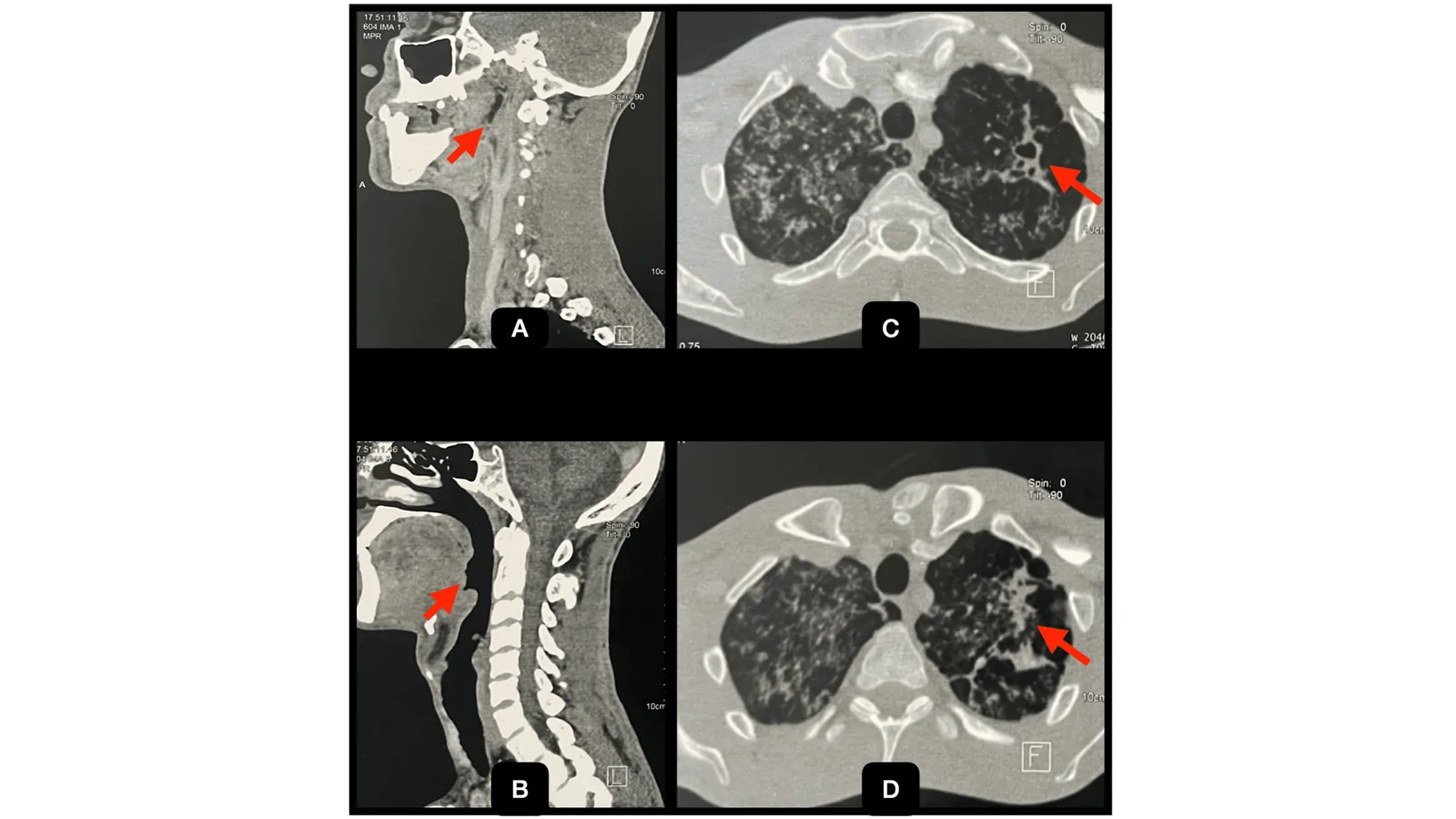
Contrast-enhanced cervicothoracic CT scan demonstrating pulmonary and tonsillar involvement (A-B) Oropharynx CT findings: tonsillar irregularity lining the palatine tonsils and epiglottic edema. (C-D) Pulmonary CT findings: bilateral apical pulmonary micronodules and a solid nodule associated with a cavitary lesion. CT: computed tomography

To further characterize these abnormalities, contrast-enhanced cervicothoracic computed tomography (CT) was performed. Cervical CT demonstrated irregular mucosal thickening involving both palatine tonsils, more pronounced on the right side, associated with edema of the epiglottis and the glossoepiglottic folds. Thoracic CT revealed bilateral apical micronodular miliary opacities, a 15-mm solid pulmonary nodule, multiple cavitary lesions, and parenchymal calcifications within the left apicodorsal segment (Figure 2).

A sputum GeneXpert MTB/RIF assay detected *Mycobacterium tuberculosis* with a high bacillary load and no evidence of rifampicin resistance, thereby confirming the diagnosis of pulmonary tuberculosis.

**Figure 3:**
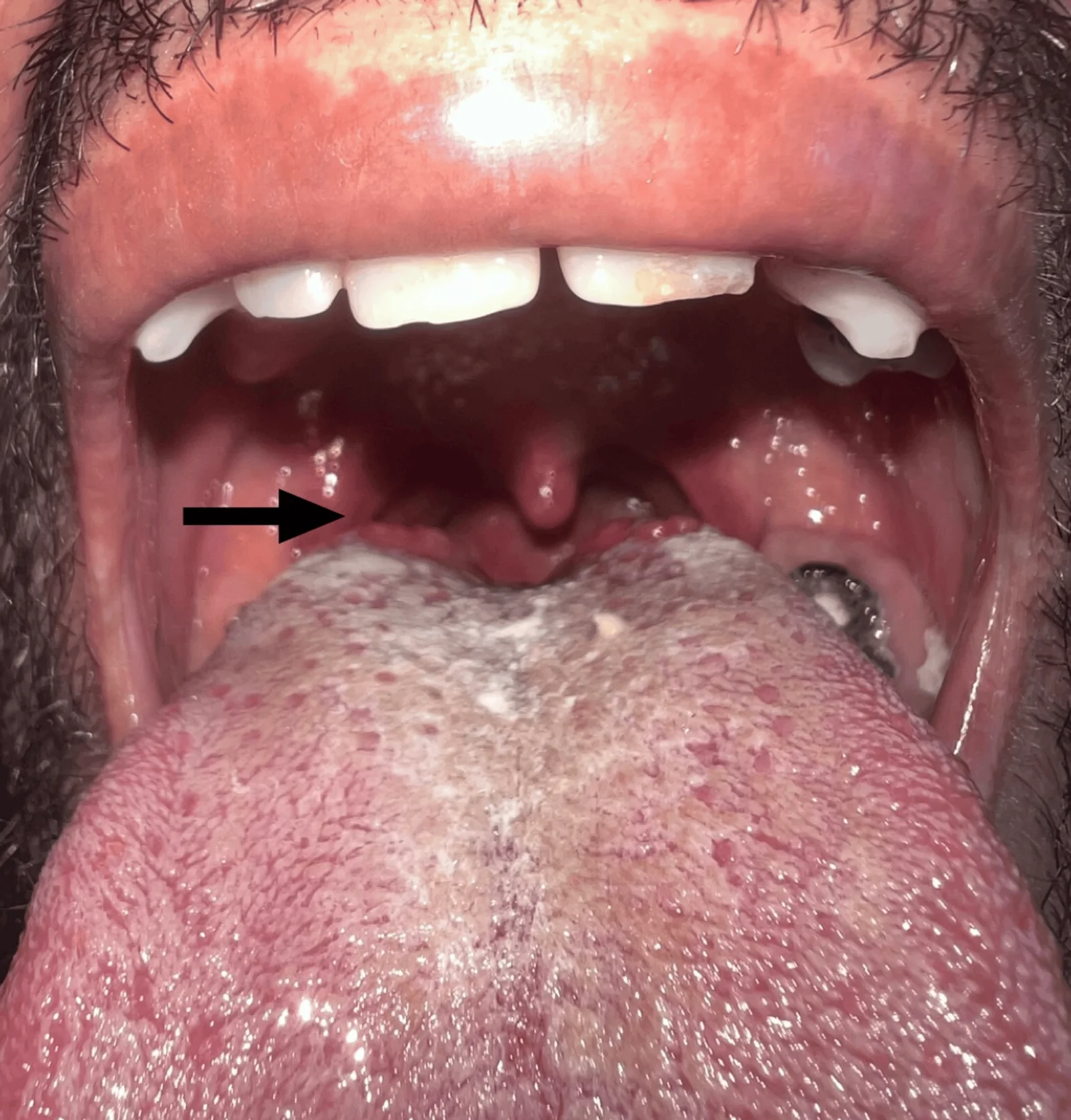
Oropharyngeal examination showing right tonsillar hypertrophy

Subsequent otorhinolaryngological examination revealed poor oral hygiene without limitation of mouth opening, inflammatory enlargement of the anterior tonsillar pillar, inflammation of the pharyngeal wall, and hypertrophy of the lingual tonsil. No cervical lymphadenopathy was observed (Figure 3).

A biopsy of the palatine tonsil was performed for histopathological and microbiological examination. Histopathological examination demonstrated epithelioid and multinucleated giant cell granulomas without caseous necrosis. Microbiological culture of the palatine tonsillar biopsy specimen was positive for *Mycobacterium tuberculosis *after one month of incubation. Considering the histopathological findings, the positive tonsillar culture, and the confirmed pulmonary tuberculosis, the diagnosis of concomitant pulmonary and tonsillar tuberculosis was established.

An evaluation for underlying immunodeficiency was undertaken. Human immunodeficiency virus (HIV) serology was negative, while fasting blood glucose and glycated hemoglobin levels were within the normal range.

The patient was treated with the standard six-month anti-tuberculosis regimen, consisting of a two-month intensive phase with rifampicin, isoniazid, pyrazinamide, and ethambutol (2RHZE), followed by a four-month continuation phase with rifampicin and isoniazid (4RH), in accordance with national tuberculosis treatment guidelines. Drug doses were adjusted according to the patient's body weight. Treatment adherence was regularly monitored, and no significant adverse events were reported during therapy. Clinical evolution was favorable, with progressive resolution of both respiratory and oropharyngeal symptoms.

## Discussion

Tuberculosis remains a major global health challenge, particularly in low- and middle-income countries where the disease remains endemic. Although pulmonary tuberculosis is the predominant clinical presentation, extrapulmonary tuberculosis accounts for approximately 15-20% of all reported cases and may affect virtually any organ system. Involvement of the ear, nose, and throat (ENT) region is uncommon, representing less than 2% of extrapulmonary tuberculosis cases, while tonsillar tuberculosis is exceptionally rare [1].

Tonsillar tuberculosis is usually secondary to active pulmonary disease and is believed to result from the repeated inoculation of the tonsillar mucosa by infected bronchial secretions [3,4]. The low incidence of this localization has been attributed to several protective mechanisms, including the bactericidal properties of saliva, the resistance of the stratified squamous epithelium, the cleansing action of swallowing, and the normal oral microbial flora. Conditions such as poor oral hygiene, chronic smoking, malnutrition, immunosuppression, or mucosal injury may disrupt these protective barriers and predispose patients to tonsillar infection [4].

The clinical presentation of tonsillar tuberculosis is nonspecific. The most common symptoms include persistent odynophagia, dysphagia, sore throat, cervical lymphadenopathy, and unilateral or bilateral tonsillar enlargement. These findings frequently mimic malignant tumors of the oropharynx, particularly squamous cell carcinoma, leading to delayed diagnosis [5]. Consequently, any atypical tonsillar lesion occurring in a patient from a tuberculosis-endemic area should prompt the consideration of tuberculosis in the differential diagnosis, while malignancy must always be excluded.

Chest imaging remains an important component of the diagnostic workup, particularly because tonsillar tuberculosis, although rare, may occur secondary to active pulmonary tuberculosis. In patients presenting with suspected tonsillar tuberculosis, chest imaging can therefore help identify concomitant pulmonary involvement [6]. In our patient, chest radiography and CT demonstrated bilateral cavitary and infiltrative lesions highly suggestive of pulmonary tuberculosis. Molecular testing using the GeneXpert MTB/RIF assay rapidly confirmed the diagnosis by detecting *Mycobacterium tuberculosis *without rifampicin resistance [7]. The introduction of nucleic acid amplification tests has considerably improved the early diagnosis of tuberculosis by providing the rapid detection of *Mycobacterium tuberculosis *and the simultaneous identification of rifampicin resistance [1].

Histopathological examination may provide important supportive evidence for tonsillar tuberculosis. Typical findings include epithelioid granulomas with multinucleated giant cells and caseous necrosis. However, these findings are not specific, particularly in the absence of caseous necrosis. In our patient, the histopathological findings were supported by microbiological examination of the tonsillar biopsy, which confirmed the presence of *Mycobacterium tuberculosis*, as well as by the compatible radiological findings [7].

The treatment of tonsillar tuberculosis does not differ from that of pulmonary tuberculosis. Standard anti-tuberculosis chemotherapy, consisting of a two-month intensive phase with rifampicin, isoniazid, pyrazinamide, and ethambutol (2RHZE), followed by a four-month continuation phase with rifampicin and isoniazid (4RH), remains highly effective in drug-susceptible disease. Surgical intervention is generally limited to obtaining tissue for histopathological diagnosis or managing rare local complications [8].

The coexistence of pulmonary and tonsillar tuberculosis remains exceptionally uncommon in the literature. This case emphasizes the importance of performing a comprehensive ENT examination in patients presenting with pulmonary tuberculosis and upper aerodigestive symptoms. Conversely, pulmonary tuberculosis should be investigated in patients presenting with unexplained tonsillar lesions when clinical, epidemiological, or histopathological findings raise suspicion, particularly in endemic settings. Early diagnosis and prompt initiation of anti-tuberculosis therapy are essential to prevent disease progression, reduce transmission, and avoid unnecessary invasive procedures.

## Conclusions

The coexistence of pulmonary and tonsillar tuberculosis is an exceptionally rare clinical entity that may pose significant diagnostic challenges because of its nonspecific clinical presentation and resemblance to oropharyngeal malignancies. Clinicians should maintain a high index of suspicion, particularly in tuberculosis-endemic regions, when evaluating atypical tonsillar lesions associated with constitutional or respiratory symptoms. A multidisciplinary approach combining clinical assessment, thoracic imaging, microbiological investigations, and histopathological examination is essential for establishing an accurate diagnosis. Early recognition and prompt initiation of standard anti-tuberculosis therapy can lead to favorable outcomes while preventing disease progression and unnecessary invasive procedures.
